# Blood Pressure Changes in Association with Nimodipine Therapy in Patients with Spontaneous Subarachnoid Hemorrhage

**DOI:** 10.1007/s12028-023-01760-y

**Published:** 2023-06-12

**Authors:** Verena Rass, Philipp Kindl, Anna Lindner, Mario Kofler, Klaus Altmann, Lauma Putnina, Bogdan-Andrei Ianosi, Alois J. Schiefecker, Ronny Beer, Bettina Pfausler, Raimund Helbok

**Affiliations:** 1 Neurological Intensive Care Unit, Department of Neurology, Medical University of Innsbruck, Anichstrasse 35, 6020 Innsbruck, Austria; 2 Department of Neurology, Kepler University Hospital, Johannes Kepler University Linz, Linz, Austria

**Keywords:** Subarachnoid hemorrhage, Nimodipine, Arterial hypotension, Hemodynamics, Delayed cerebral ischemia

## Abstract

**Background:**

Nimodipine is recommended to prevent delayed cerebral ischemia in patients with spontaneous subarachnoid hemorrhage (SAH). Here, we studied hemodynamic side effects of different nimodipine formulations (per os [PO] and intravenous [IV]) in patients with SAH undergoing continuous blood pressure monitoring.

**Methods:**

This observational cohort study includes consecutive patients with SAH (271 included in the IV group, 49 in the PO group) admitted to a tertiary care center between 2010 and 2021. All patients received prophylactic IV or PO nimodipine. Hemodynamic responses were evaluated based on median values within the first hour after continuous IV nimodipine initiation or PO nimodipine application (601 intakes within 15 days). Significant changes were defined as > 10% drop in systolic blood pressure (SBP) or diastolic blood pressure from baseline (median values 30 min before nimodipine application). With the use of multivariable logistic regression, risk factors associated with SBP drops were identified.

**Results:**

Patients were admitted with a median Hunt & Hess score of 3 (2–5; IV 3 [2–5], PO 1 [1–2], *p* < 0.001) and were 58 (49–69) years of age. Initiation of IV nimodipine was associated with a > 10% SBP drop in 30% (81/271) of patients, with a maximum effect after 15 min. A start or increase in noradrenaline was necessary in 136/271 (50%) patients, and colloids were administered in 25/271 (9%) patients within 1 h after IV nimodipine initiation. SBP drops > 10% occurred after 53/601 (9%) PO nimodipine intakes, with a maximum effect after 30–45 min in 28/49 (57%) patients. Noradrenaline application was uncommon (3% before and 4% after nimodipine PO intake). Hypotensive episodes to an SBP < 90 mm Hg were not observed after IV or PO nimodipine application. In multivariable analysis, only a higher SBP at baseline was associated with a > 10% drop in SBP after IV (*p* < 0.001) or PO (*p* = 0.001) nimodipine application, after adjusting for the Hunt & Hess score on admission, age, sex, mechanical ventilation, days after intensive care unit admission, and delayed cerebral ischemia.

**Conclusions:**

Significant drops in SBP occur in one third of patients after the start of IV nimodipine and after every tenth PO intake. Early recognition and counteracting with vasopressors or fluids seems necessary to prevent hypotensive episodes.

**Supplementary Information:**

The online version contains supplementary material available at 10.1007/s12028-023-01760-y.

## Introduction

Delayed cerebral ischemia (DCI) occurs in up to 20–30% of patients with spontaneous subarachnoid hemorrhage (SAH) and continues to be one of the most important factors contributing to poor long-term outcome, especially if it leads to cerebral infarction [1, 2]. Traditionally, large-vessel vasospasm has been thought to be the main cause of DCI; however, there is increasing evidence supporting a multifactorial cause of DCI including impaired microcirculation, neuroinflammation, microvasospasm and macrovasospasm, cortical spreading depolarization/ischemia, microthromboembolism, impaired autoregulation, and others [2].

To date, the only recommended drug for the prevention of DCI is nimodipine, an L-type dihydropyridine calcium channel antagonist [3, 4]. It reduces the risk of DCI and poor functional outcome after SAH [5–7]. Mechanisms of action are likely more complex than simple inhibition of vasoconstriction and include reduction of vasospasm, neuroprotection, enhancement of fibrinolytic activity and thus reduction of microthrombism [8], and diminishment of cortical spreading depolarizations [9, 10]. Nimodipine is available as oral capsules (in the United States), oral tablets (in Europe), oral solutions, and intravenous (IV) infusion solution (in Europe). International guidelines endorse the application of Food and Drug Administration and European Medicines Agency–approved oral nimodipine.

Systemic arterial hypotension remains the most important adverse effect of nimodipine treatment [11, 12]. This is of special concern in hemodynamically unstable patients with SAH, and counteractive measures including the use of vasopressors, application of fluids, or a reduction in nimodipine dosage may be necessary. Importantly, profound hypotensive episodes are associated with brain hypoperfusion, brain tissue hypoxia, and other mechanisms of secondary brain injury and may ultimately contribute to brain ischemia and poor neurological outcome in patients after SAH [13, 14].

Interestingly, clinical trials testing a formulation of nimodipine do not consistently report adverse events of hypotension, and there is little real-life clinical data describing a temporal relation of per os (PO) and IV nimodipine application and blood pressure changes [11]. Moreover, early drops in blood pressure may not be recognized in patients with discontinuous blood pressure monitoring.

In the current study, we report the prevalence of significant blood pressure drops and the development of relevant hypotensive episodes following IV and PO nimodipine in consecutive patients with SAH undergoing continuous blood pressure monitoring through an arterial line. Following our internal protocol, IV nimodipine was applied as continuous infusion in patients with poor-grade SAH, whereas awake patients without swallowing difficulties received PO nimodipine. We hypothesized that blood pressure drops are common after nimodipine application, prompting treatment intensification with vasopressors or fluids.

## Methods

### Study Design, Setting, and Patient Selection

The study design was guided by the STROBE statement on observational studies. This is a retrospective analysis of prospectively collected data of patients with nontraumatic SAH who were admitted to the neurological intensive care unit (ICU) of a tertiary care hospital (Medical University of Innsbruck) between 2010 and 2021. Inclusion criteria were (1) diagnosis of spontaneous SAH confirmed by computed tomography (CT) scan or lumbar puncture irrespective of whether an aneurysm was found; (2) male or female sex and 18 years of age or older; (3) ICU stay for more than 24 h; (4) invasive, continuous blood pressure monitoring to achieve high granularity (5 min) of hemodynamic variables (blood pressure, heart rate [HR]) at the time of nimodipine application (IV or PO). Nineteen patients adhering to the IV nimodipine group were excluded because IV nimodipine was started simultaneously with continuous blood pressure measurement, leading to missing baseline blood pressure values. For calculations of hemodynamics secondary to PO nimodipine administration, only patients in whom PO nimodipine was administered before IV nimodipine were considered (i.e., patients with a switch from IV to PO have been excluded from the PO group). According to our protocol, PO formulations may be given in poor-grade patients before a central venous catheter is installed, followed by a switch to IV nimodipine thereafter. De-escalation from IV to PO nimodipine is usually performed after vasospasm has resolved more than 2 weeks after aneurysmal bleeding. Therefore, none of our analysis with PO formulations occurred after the cessation of IV nimodipine.

The conduct of the study was approved by the local ethics committee (Medical University of Innsbruck, AM4091-292/4.6). Written informed consent was obtained according to local regulations.

### Patient Management and Grading

Patients were treated according to international guidelines [3, 4]. Clinical grading was done with use of the Hunt & Hess (H&H) score [15]. Admission CT scans were scored using the modified Fisher score [16]. Ruptured aneurysms were secured by neurosurgical clipping or endovascular coiling after an interdisciplinary discussion between neurologists, neuroradiologists, and neurosurgeons. Transcranial color-coded duplex sonography (TCCS) (LOGIQ S8; GE Healthcare, Chicago, IL) was performed on a regular basis to screen patients for large-vessel vasospasm. Sonographic vasospasm was defined as an elevation of mean velocities greater than 120 cm/s in the middle or anterior cerebral artery or a daily change in mean TCCS velocities greater than 50 cm/s. Severe vasospasm (> 200 cm/s) was further confirmed by catheter cerebral angiogram. DCI was defined by a clinical deterioration with a new focal neurologic deficit, a decrease of greater than or equal to two points on the Glasgow Coma Scale, or a new infarct on the CT or magnetic resonance imaging scan not attributable to other causes [17]. In unconscious patients, deterioration of multimodal neuromonitoring parameters (brain tissue oxygen tension, cerebral metabolism) were also considered for the diagnosis of DCI [2]. Severe vasospasm or DCI was treated with induced hypertension. In case of persistent neurological deficits or pathologic levels acquired by multimodal neuromonitoring, cerebral panangiography was pursued to evaluate for intraarterial nimodipine treatment.

All patients received prophylactic nimodipine as of admission to the ICU. Nimodipine was applied intravenously in poor-grade patients and orally in good-grade patients based on clinical, laboratory, and radiographic parameters. In some poor-grade patients, oral nimodipine was switched to IV nimodipine as soon as they had a central line. Continuous IV nimodipine (Nimotop 10 mg; Bayer Austria, Wien, Austria) perfusion was started at a testing dosage of 1 mg/h and increased to 2 mg/h if it was hemodynamically tolerated. The targeted daily dose was 48 mg. Dosage reductions were done in the setting of refractory hypotension despite vasopressor escalation. The full dose of 60 mg of oral nimodipine (tablet, Nimotop 30 mg; Bayer Austria) was given six times a day resulting in a targeted daily dose of 360 mg, as used in the original protocol [6]. Deviating from the original protocol, nimodipine was started as early as possible [6]. If not tolerated, the dosage was reduced to 30 mg every 4 h. Usually, nimodipine was given for 2 weeks and tapered thereafter based on whether large-vessel vasospasm was still evident or not. Blood pressure drops secondary to nimodipine application were treated with fluid boluses or vasopressors, mainly noradrenaline.

Minimum mean arterial blood pressure (MAP) levels targeted for were > 65 mm Hg. However, blood pressure targets were adapted to the clinical course (i.e., DCI) and monitoring parameters, if available, to achieve optimal cerebral perfusion. For hemodynamic augmentation, vasopressors or fluids were used, as decided by the treating physician.

### Data Collection

Patients’ demographics, hospital complications, and outcomes were prospectively collected and discussed in weekly meetings held by the study team and treating neurointensivists.

Continuous variables including systolic blood pressure (SBP), diastolic blood pressure (DBP), and HR were saved in a granularity of 3 min using a patient data management system (CentricityTM Critical Care 8.1 SP7; GE Healthcare Information Technology, Dornstadt, Germany) and calculated as 5-min medians for the purposes of this study. Arterial blood pressure was measured through either radial or, rarely, femoral arterial lines, and HR was either measured through an arterial line or was electrocardiogram-based. Exact timings and doses of nimodipine administered to patients with SAH were also saved in the server of the patient data management system. Functional neurological outcome was assessed at 3 months via telephone interview by a study nurse blinded to the disease course and rated with the modified Rankin Scale score (mRS). Twenty-three patients were lost to follow-up.

### Study Outcome Measures

The primary outcome measure was the prevalence of drops in blood pressure (> 10% of median SBP or DPB) within 1 h after IV or PO nimodipine application in relation to median baseline blood pressures (30 min before nimodipine application). Further, we evaluated the prevalence of HR increases (> 10%), new onset hypotension (SBP ≤ 90 mm Hg), bradycardia (< 50 beats per minute), and tachycardia (> 100 beats per minute) within 1 h after IV or PO nimodipine application.

### Data Management and Statistical Analysis

The first 15 days of ICU admission were defined as the study duration. The first 24 h after ICU admission were denoted as day 0.

Abnormal hemodynamic values were checked manually to eliminate artifacts. Unreliable systolic (< 40 mm Hg or > 250 mm Hg) and diastolic (< 15 mm Hg or > 180 mm Hg) blood pressure values secondary to transducer flushing, movement artifacts, connection-reconnection artifacts, or obstructed arterial lines were deleted.

Variables are shown as counts (%), median and interquartile range or mean ± standard deviation, as appropriate. Continuous variables were tested for normality and compared using the Student’s *t*-test or Mann–Whitney *U*-test. Differences between binary variables were analyzed using the Fisher’s exact test. For oral nimodipine, all intakes within the study period of 15 days were considered whenever simultaneous blood pressure readings were available. For IV nimodipine, only the starting and the first dosage increase (1 mg/h to 2 mg/h) were considered for statistical analysis. Clinically significant changes of hemodynamics (10%) were calculated based on the difference between baseline (median SBP, DBP, and HR 30 min before nimodipine application) and an observation period of 1 h (median values) after nimodipine application. Separate analysis was done for prespecified disease phases (days 0–2, days 3–5, days 6–10, days 11–14) for PO nimodipine. Correlations between drops in SPB, DPB, and HR were calculated with Spearman rho.

Multivariable logistic regression analysis with use of generalized linear models (IV group) or generalized estimating equation models with an autoregressive correlation matrix (AR(1)) to account for repeated measures (PO group) was done to find risk factors associated with significant SBP drops secondary to nimodipine delivery. Multivariable models were adjusted for important predefined covariates (disease severity as measured by H&H score on admission, age, sex, baseline SBP, mechanical ventilation, days after ICU admission, and DCI).

A *p* value less than 0.05 was considered statistically significant. Statistical analysis was performed using IBM SPSS Statistics version 24 64-bit edition.

## Results

### Study Population

Based on the selection of patients with SAH undergoing continuous blood pressure monitoring, 271 out of 425 patients qualified for the IV nimodipine analysis, and 49 patients were included in the PO nimodipine group; eight patients qualified for both groups as PO and then IV nimodipine were used in sequence (Supplemental Fig. 1).

Patients were admitted with a median H&H score of 3 (2–5; IV 3 [2–5], PO 1 [1–2], *p* < 0.001) and were 58 (49–69) years of age (Table 1). Large-vessel vasospasm was observed in 50% of patients either based on TCCS and/or angiography (*n* = 160; IV: 55%, PO: 25%, *p* < 0.001) and 19% (*n* = 60; IV: 21%, PO:4%, *p* = 0.002) developed DCI. Secondary to lower disease severity and hospital complications, hospital mortality was lower in the PO group (2%, *n* = 1) as compared with the IV group (12%, *n* = 31, *p* = 0.040).

**Table 1:** Patient demographics, baseline characteristics, hospital complications, and outcomes

Parameter	Oral nimodipine (*n* = 49)	IV nimodipine (*n* = 271)	*p* value^a^
Baseline characteristics			
Age	53 (48–65)	58 (49–79)	0.145
Female sex	24 (49)	181 (67)	0.023
Admission variables			
Admission Hunt & Hess score	1 (1–2)	3 (2–5)	< 0.001
1	26 (53)	50 (19)	
2	18 (37)	55 (20)	
3	3 (6)	57 (21)	
4	1 (2)	28 (10)	
5	1 (2)	81 (30)	
Modified Fisher score on admission	2 (1–3)	4 (3–4)	< 0.001
Aneurysm treatment			
Coiling	19 (39)	164 (61)	< 0.001
Clipping	4 (8)	80 (30)	
No intervention	25 (51)	22 (8)	
Withhold therapy	1 (2)	5 (2)	
Hospital complications			
Hydrocephalus requiring external ventricular drain	3 (6)	176 (65)	< 0.001
Mechanical ventilation during ICU stay	16 (33)	254 (94)	< 0.001
Large-vessel vasospasm	12 (25)	148 (55)	< 0.001
Delayed cerebral ischemia	2 (4)	58 (21)	0.002
Sepsis/bacteremia	4 (8)	50 (19)	0.096
Pneumonia	4 (8)	128 (47)	< 0.001
Ventriculitis	2 (4)	35 (13)	0.090
Urinary tract infection	10 (20)	31 (12)	0.477
Outcomes			
Hospital mortality	1 (2)	31 (12)	0.040
3-month functional outcome (modified Rankin Scale)^b^	0 (0–1)	2 (1–5)	< 0.001
0	24 (49)	35 (13)	
1	14 (29)	53 (20)	
2	2 (4)	39 (14)	
3	1 (2)	27 (10)	
4	0 (0)	22 (8)	
5	1 (2)	31 (11)	
6	1 (2)	45 (17)	

Data are given in *n* (%) or median (IQR)

*ICU* intensive care unit, *IQR* interquartile range, *IV* intravenous, *PO* per os

^a^Differences across the PO and IV group were calculated with the Mann Whitney *U*-test, Student’s *t*-test, or the Fisher's exact test, as appropriate

^b^Twenty-three patients were lost to follow-up

### Effects of IV Nimodipine on Hemodynamics and Management

Intravenous nimodipine infusion was started early after admission (median 10 [6–21] h), and most patients (79%) received a reduced testing rate of 1 mg/h initially, corresponding to treatment strategies, followed by the targeted dosage of 2 mg/h. In the remaining patients, the starting dosage was 2 mg/h. The full daily nimodipine dose (48 mg) was administered during 28% of study days, and at least 80% of the targeted daily nimodipine doses (38.4 mg) were reached in 54% of the study days. Ninety-three percent of patients received noradrenaline during the study period (Table 2). The requirement of noradrenaline increased from 52% (1 h before) to 75% within 1 h after nimodipine initiation (Table 2). Treatment intensification in terms of an increase in the noradrenaline dose (> 10%) or the start of noradrenaline therapy was necessary in 136/271 (50%) patients within 1 h after nimodipine start. There was no difference in the presence or absence of significant blood pressure drops across the groups (*p* = 0.146, Supplemental Table 1). Colloids were administered in 25/271 (9%) patients within 1 h after starting IV nimodipine.

**Table 2:** Nimodipine therapy and reactive treatment adaptions

Parameter	Oral nimodipine (*n* = 49, 601 intakes)	IV nimodipine (*n* = 271)
Hours between ICU admission and initiation of nimodipine therapy	10 (6–15)	10 (6–21)
Duration of nimodipine therapy within study period, days^a^	10 (7–13), change to IV possible	14 (12–15)
Days without vasopressors (noradrenaline) during nimodipine therapy within study period, days	9 (7–12) in 49 patients	8 (5–11) in 224 patients
Days with vasopressors (noradrenaline) during nimodipine therapy within study period, days	2 (1–3) in 11 patients	6 (3–10) in 248 patients
Percentage of 80% of daily nimodipine dose application (288 mg PO/38.4 mg IV) within the study period^b^	218/398 days (45%), 29/49 patients (60%), any day within study period, change to IV possible	1622/3009 days (54%), 226/271 patients (83%), any day within study period
Percentage of full daily nimodipine dose (360 mg PO/48 mg IV) within the study period^b^	99/398 days (25%), 17/48 patients (35%), any day within study period, change to IV possible	847/3009 days (28%), 205/271 patients (76%), any day within study period
Hours between ICU admission and initiation of noradrenaline	8 (5–23)	5 (1–8)
Noradrenaline within study period	13/49 (27)	251/271 (93)
Use of noradrenaline at baseline (1 h before)	18/601 (3)	141 (52)
Use of noradrenaline after nimodipine application (within 1 h)	23/601 (4)	202 (75)
Dose of vasopressors before nimodipine application (1 h before), mg	0.138 (0.069–0.348)	0.233 (0.102–0.500)
Dose of vasopressors after nimodipine application (within 1 h), mg	0.166 (0.074–0.590)	0.295 (0.185–0.525)

Data are given in *n* (%) or median (IQR)

*ICU* intensive care unit, *IQR* interquartile range, *IV* intravenous, *PO *per os

^a^Irrespective of concomitant continuous blood pressure measurements

^b^Days when no IV or PO nimodipine was given were not included

**Fig. 1:**
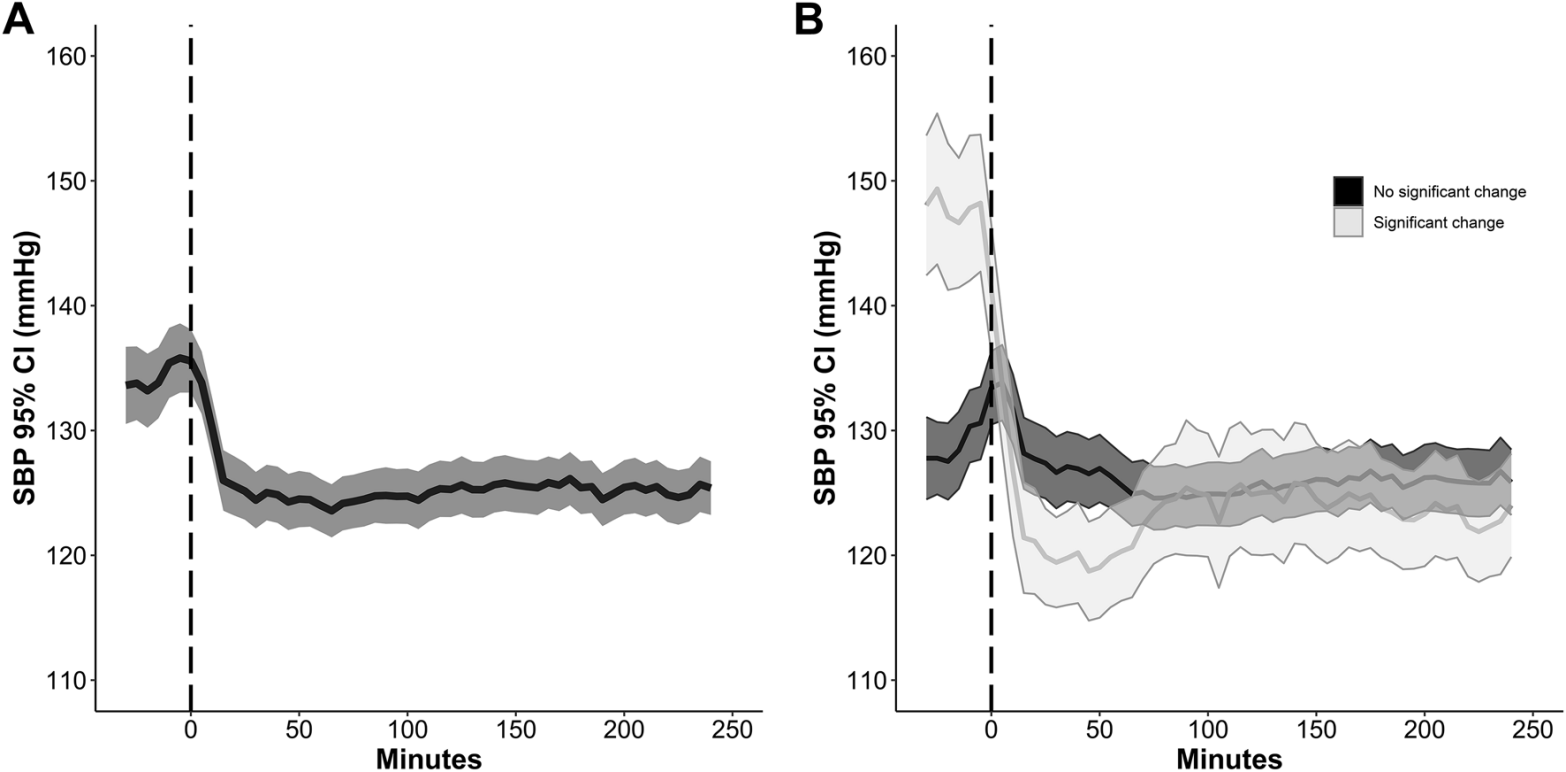
Panel **a** reports the evolution over time of the median (95% CI) systolic blood pressure (SBP) after initiation of IV nimodipine in 271 patients. **b** Median SBP over time after initiation of IV nimodipine in the subgroups of patients with (*n* = 81, 30%) and without a significant > 10% SBP drop. The 0 refers to the start of continuous nimodipine application. Values and bars are median values over 5 min, reported every 5 min. CI, confidence interval, IV, intravenous

Initiation of IV nimodipine was associated with a median SBP decrease of 8 mm Hg from 134 ± 22 mm Hg at baseline to 126 ± 18 mm Hg (Fig. 1, Table 3), resulting in a significant SBP drop > 10% in 30% (81/271) of patients, with a maximum decrease after 15 min. The prevalence of significant SBP drops was higher when starting at 2 mg/h (55%) as compared with the reduced starting dosage (drops in 23%). An SBP drop > 20 mm Hg from baseline was uncommon (4%) and none of the patients developed hypotension (new onset SBP ≤ 90 mm Hg) within 1 h after continuous IV nimodipine initiation.

**Table 3:** Hemodynamics within 30 min before (baseline) and within 1 h after IV or PO nimodipine application

Parameter	Oral nimodipine (*n* = 49, 601 intakes)	IV nimodipine (*n* = 271)
Baseline SBP, mm Hg	137 ± 18	134 ± 22
1 h SBP, mm Hg	136 ± 17	126 ± 18
Baseline DBP, mm Hg	64 ± 9	62 ± 11
1 h DBP, mm Hg	63 ± 9	57 ± 9
Baseline HR, bpm	68 ± 12	69 ± 16
1 h HR, bpm	69 ± 12	69 ± 15

Data are given in mean ± SD

*DBP* diastolic blood pressure, *HR* heart rate, *IV* intravenous, *PO* per os, *SBP* systolic blood pressure, *SD *standard deviation

**Fig. 2:**
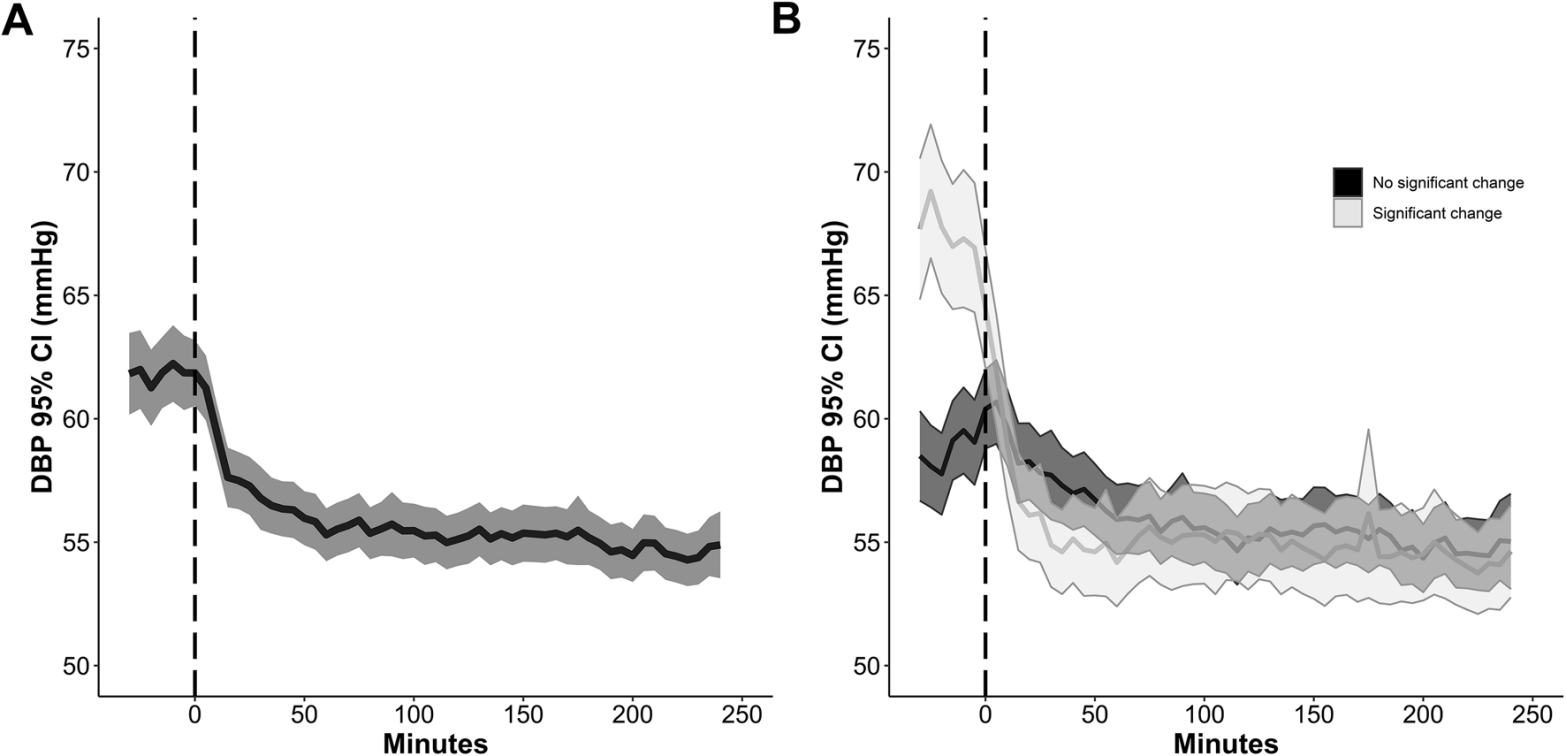
Panel **a** reports the evolution over time of the median (95% CI) diastolic blood pressure (DBP) after initiation of IV nimodipine in 271 patients. **b** Median DBP over time after initiation of IV nimodipine in the subgroups of patients with (*n* = 97, 36%) and without a significant > 10% DBP drop. The 0 refers to the start of continuous nimodipine application. Values and bars are median values over 5 min, reported every 5 min. CI, confidence interval, IV, intravenous

Significant DBP drops > 10% were observed in 36% (97/270; Fig. 2) and a DBP drop > 10 mm Hg from baseline was only seen in two (0.7%) patients.

HR increases > 10% (17%) were as frequent as decreases > 10% (17%). New onset tachycardia or bradycardia were evident in 1.5% and 4%, respectively within 1 h after nimodipine initiation.

The first increase of the IV nimodipine dosage (1 to 2 mg/h) was performed after 44 (12–97) hours in 180 patients and was associated with a SBP drop from 148 to 143 mm Hg. Fourteen percent of patients (25/180) developed a significant SBP drop > 10% at this time. Supplemental Fig. 2 demonstrates a subanalysis of dynamics of brain tissue oxygen tension (P_bt_O_2_) values before and after initiation of IV nimodipine in a limited number of 22 patients.

### Effects of PO Nimodipine on Hemodynamics and Management

The median number of oral nimodipine intakes with available data on continuous blood pressure monitoring was 10 (4–16) per patient, resulting in 601 intakes analyzed within the study time. Of the 601 intakes, 577 (96%) corresponded to nimodipine at full 60 mg dose, and 24 (4%) at the reduced dose of 30 mg. The full daily nimodipine dose (360 mg) was delivered in 25% of study days and at least 80% of the targeted nimodipine daily dose (288 mg) was reached in 45% of the study days (Table 2). A total of 13 patients (27%) received noradrenaline at any time during the study period. Among the 601 intakes analyzed, noradrenaline was already administered 1 h before nimodipine therapy in 3% (*n* = 18), and in 4% (*n* = 23) noradrenaline was given within 1 h after nimodipine administration (Table 2, Supplemental Table 1).

**Fig. 3:**
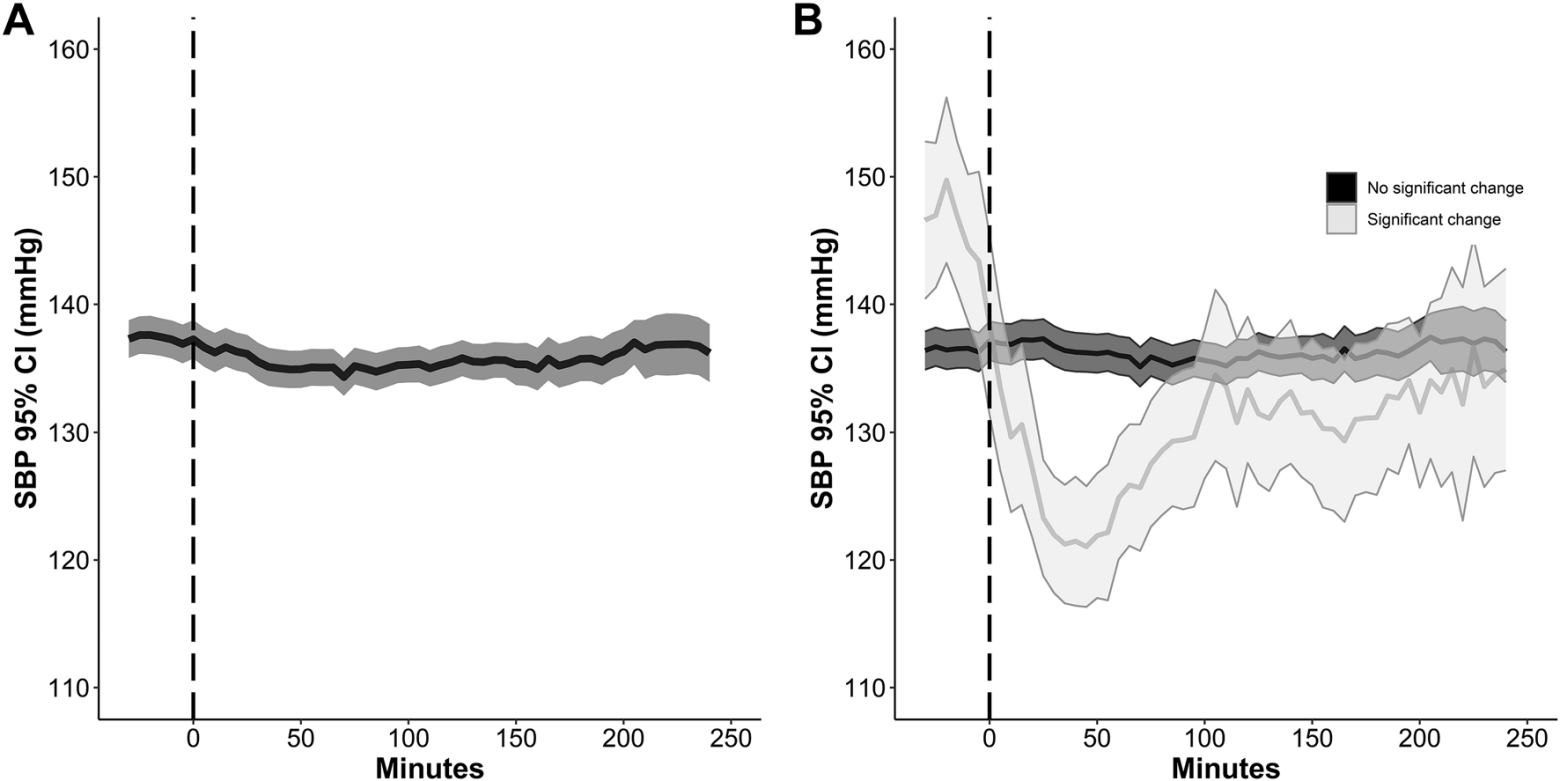
Panel **a** reports the evolution over time of the median (95% CI) systolic blood pressure (SBP) after 601 intakes of oral nimodipine in 49 patients. **b** Median SBP over time after PO nimodipine intake in the subgroups of episodes with (*n* = 53, 9%) and without a significant > 10% SBP drop. At time 0, data were available from 601 (100%) episodes. After 60 min, 120 min, 180 min, and 240 min, data were available from 590 (98%), 579 (96%), 435 (72%), and 268 (45%) intakes, respectively (and therefore not overlapping with the next dose of oral nimodipine intake). The 0 refers to the start of continuous nimodipine application. Values and bars are median values over 5 min, reported every 5 min. CI, confidence interval, PO, per os

**Fig. 4:**
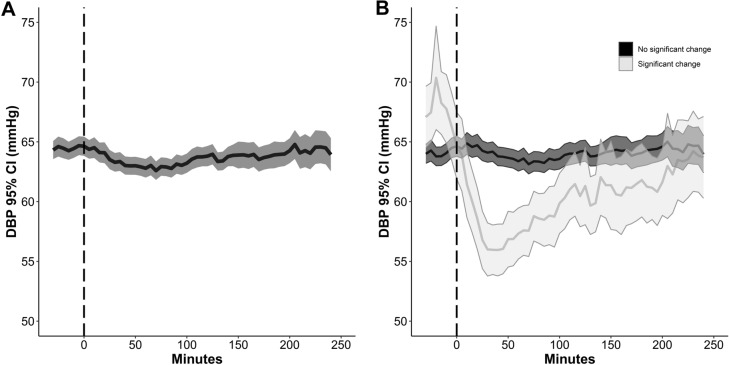
Panel **a** reports the evolution over time of the median (95% CI) diastolic blood pressure (DBP) after 601 intakes of oral nimodipine in 49 patients. **b** Median DBP over time after PO nimodipine intake in the subgroups of intakes with (*n* = 62, 10%) and without a significant > 10% DBP drop. At time 0, data were available from 601 (100%) episodes. After 60 min, 120 min, 180 min, and 240 min, data were available from 590 (98%), 579 (96%), 435 (72%), and 268 (45%) intakes, respectively (and therefore not overlapping with the next dose of oral nimodipine intake). The 0 refers to the start of continuous nimodipine application. Values and bars are median values over 5 min, reported every 5 min. CI, confidence interval, PO, per os

Overall, oral nimodipine had minor effects on blood pressure; SBP was 137 ± 18 mm Hg at baseline and 136 ± 17 mm Hg within 1 h after nimodipine intake (Figs. 3 and 4, Table 3). However, the main outcome (> 10% drop in SBP) was evident after 53/601 (9%) intakes and the maximum effect was observed 30 to 45 min post oral nimodipine administration (Figs. 3 and 4). Still, 28/49 (57%) patients with SAH experienced at least one episode of SBP drop within 1 h after PO nimodipine intake during the study period of 15 days. Similar results were obtained when intakes were stratified by different time periods post bleeding (> 10% SBP drop, days 0–2, 10%; days 3–5, 6%; days 6–10, 8%). An SBP drop > 20 mm Hg from baseline was rarely observed (19/601, 3%). Oral nimodipine application did not lead to new onset SBP ≤ 90 mm Hg.

Similarly, a significant > 10% drop in DBP was observed in 62/601 (10%) intakes corresponding to 32/49 patients (65%) who experienced at least one episode of DBP drop after PO nimodipine within the study period of 15 days. An absolute DBP drop > 10 mm Hg from baseline was observed after 27/601 (5%) intakes.

HR increased > 10% in 56 (9%) out of 598 intakes (Supplemental Fig. 3) with a maximum effect observed at about 30 min post intake. New onset tachycardia was observed only in 3/598 (0.5%) episodes.

Only weak correlations between SBP drops and HR increases, or DBP drops and HR increases, were observed at rho = 0.117 and rho = 0.119, respectively. The correlation between SBP drops and DBP drops was moderate (rho = 0.493).

### Risk Factors Associated with Significant SBP Drops > 10%

In multivariable analysis adjusted for the H&H score, age, sex, mechanical ventilation, days after ICU admission, and DCI, only a higher SBP median value at baseline was significantly associated with a consecutive drop in SBP after IV (*p* < 0.001) or PO (*p* = 0.001) nimodipine application (Table 4, Supplemental Table 2).

**Table 4:** Multivariable models identifying risk factors for > 10% drops in systolic blood pressure within 1 h after IV or PO nimodipine application

Patient group	Adjusted OR	95% CI	*p* value
IV group			
Admission Hunt & Hess score	0.961	0.773–1.193	0.716
Female sex	1.283	0.665–2.473	0.457
Age, years	1.007	0.985–1.029	0.530
Mechanical ventilation	1.703	0.803–3.612	0.165
Days after ICU admission	0.855	0.629–1.162	0.317
Delayed cerebral ischemia	1.263	0.610–2.617	0.529
Median SBP at baseline, mm Hg^a^	1.060	1.042–1.079	< 0.001*
PO group			
Admission Hunt & Hess score	0.817	0.548–1.219	0.322
Female sex	1.665	0.819–3.384	0.159
Age, years	1.018	0.987–1.050	0.251
Mechanical ventilation	1.738	0.472–6.397	0.406
Days after ICU admission	0.981	0.827–1.165	0.829
Delayed cerebral ischemia	1.064	0.158–7.183	0.949
Median SBP at baseline, mm Hg^a^	1.034	1.014–1.055	0.001*

Multivariable logistic regression analysis was done with generalized linear models (IV group) or generalized estimating equation models with an autoregressive correlation matrix (AR-1) to account for repeated measures (PO group)

*CI* confidence interval, *ICU* intensive care unit, *IV* intravenous, *OR* odds ratio, *PO* per os, *SBP* systolic blood pressure

^a^Thirty minutes before nimodipine application

*Statistically significant *p* <0.05

### Effects of Nimodipine Dose on Hospital Complications and Outcome

The median daily nimodipine dose had no independent effect on the occurrence of vasospasm (PO *p* = 0.137; IV *p* = 0.308), DCI (IV *p* = 0.076) or poor functional 3-month outcome (mRS > 2; IV *p* = 0.344) after adjusting for the H&H score and age. Out of the PO nimodipine group, only 4% and 7% of patients were diagnosed with DCI or poor functional outcome, respectively, thus making statistical analysis unreliable.

## Discussion

In this observational study we found that clinically significant blood pressure drops occurred in every third patient with SAH after continuous IV nimodipine start and after every tenth oral nimodipine intake. These changes were counteracted by vasopressors or IV fluids in most instances. Systemic hypotension ≤ 90 mm Hg was not recorded. The maximal effect on blood pressure was observed 15 (IV) and 45 (PO) minutes after nimodipine application. These results strongly argue for continuous blood pressure monitoring in patients with SAH.

Our data reflect clinical real-life situation comprising a large population of patients with SAH including all severity grades, where continuous blood pressure monitoring was applied. The high granularity of blood pressure readings and the time-locked application of nimodipine allowed a precise analysis of blood pressure changes during the study period. Decreases of > 10% in SBP were evident in every third patient after a starting dosage of 1 mg/h of IV nimodipine. Post oral nimodipine intake, blood pressure drops occurred in 10%, irrespective of the time period after the disease. Importantly, our protocol for PO nimodipine did not diverge from the original protocol published by Pickard et al. [6]. Different definitions of hypotension or blood pressure drops across studies [11, 18, 19] make comparisons to existing literature challenging. Based on clinical meaningfulness, we decided to define significant decreases as blood pressure drops of 10%. We cannot exclude other concurrent medications that may have influenced our results; however, simultaneous application of other drugs potentially influencing blood pressure is unlikely. In the IV nimodipine group, we focused our analysis on the initial start and timing of the first dosage increase from 1 to 2 mg/h based on the idea to report acute changes of blood pressure, as the effect is likely diluted by other drugs and potential escalations of vasopressors in the later course. Another reason, why we most likely describe the “pure” effect of nimodipine on hemodynamics is that the timely evolution of SBP drops mirror the well-known pharmacokinetic profile of nimodipine: the transient maximum effect after PO nimodipine was observed after 45 to 60 min corresponding to the Tmax of 60 mg oral nimodipine [12]. After IV nimodipine bolus injection, the initial distribution phase half-life was 7 min [20].

Importantly, we did not record hypotensive episodes ≤ 90 mm Hg post nimodipine delivery. This is most probably due to our institutional protocol targeting at least 65 mm Hg MAP in all patients with SAH with escalating strategies when DCI is diagnosed. Other reasons include vigilant monitoring and appropriate awareness with counteractive measures being rapidly applied. In this line, escalations of vasopressors were common after IV nimodipine initiation based on patients’ hemodynamic responses. Monitoring cardiac function and blood pressure is one of the important factors after SAH. It is important to notice that changes in blood pressure may have been missed if blood pressure would have been recorded discontinuously. Although we do not have a comparison group to support our hypothesis, continuous monitoring is recommended in poor-grade patients with SAH and should be extended to good-grade patients to detect significant drops in blood pressure that may counteract the neuroprotective effect of nimodipine [3, 4].

In our study, transient dosage reductions have been necessary in some patients. Literature suggests that higher doses of nimodipine may be more efficacious, however their use is limited due the risk of hypotension [10, 19]. It is important to keep in mind that systemic hypotension counteracts the concept of induced hypertension during DCI [1]. Decreases in blood pressure may prompt decreases in cerebral perfusion pressure [21], which in turn may lead to compromised brain oxygenation, especially in the setting of impaired autoregulation [21, 22]. Although studies in animals and healthy humans indicated that nimodipine increases cerebral blood flow supporting a role for L-type channels in the cerebrovascular tone [23, 24], this was not found in patients with SAH, where MAP decreases were associated with decreased cerebral blood flow [21]. This again may promote secondary brain injury. Previous studies showed that arterial hypotension occurred more often in poor-grade patients [11, 19] resulting in a higher prevalence of nimodipine dosage reductions in these patients [19]. The authors also found that nimodipine dosage reductions or discontinuations were associated with DCI and poor outcome which we could not replicate [19]. It needs to be considered that patients with poor-grade SAH more frequently need prolonged analgo-sedation, mechanical ventilation, and vasopressor support making them per-se more vulnerable to nimodipine-associated blood pressure drops as compared with good-grade patients. For clinical practice, it is reasonable to first target euvolema in the setting of a significant blood pressure drop after PO nimodipine application, and then apply a second dose in reduced dosing before discontinuation of nimodipine. For IV nimodipine, slowly titration to the target dose may be supported by adjustments in vasopressor dosages in euvolemic patients. The extend of vasopressor need is under debate and should be limited preventing the patient from harmful side effects. If additional monitoring devices (i.e., brain tissue oxygen monitoring) can exclude safety concerns, continuation of IV nimodipine may be considered even if blood pressure drops occur following an individualized approach.

Except for higher baseline blood pressure values, we could not identify any risk factors for blood pressure drops secondary to nimodipine delivery. However, these findings have to be interpreted with caution, because our patients with poor-grade SAH were mostly treated with IV and good-grade patients with PO nimodipine separating them into two distinctive groups. The higher prevalence of blood pressure drops after IV nimodipine compared to PO nimodipine may partly be explained by the aforementioned factors. Moreover, nimodipine-associated blood pressure drops were not associated with secondary neurological complications such as DCI or poor functional outcomes. This is most likely due to the overall low prevalence of hypotensive episodes which could have been counteracted by appropriate treatment.

Alternative routes of nimodipine administration with drug delivery to the most vulnerable regions minimizing systemic side effects have not yet been proven efficacious. The NEWTON study was conducted to compare the efficacy of intraventricular versus oral nimodipine [25]. It was halted in 2018 after an interim analysis because of a low probability of meeting its primary endpoint despite promising subgroup analysis suggesting that some patients may benefit from local nimodipine application [10].

Reflex tachycardia and reactive HR increases following systemic vasodilation was observed in some patients, although the correlation was only weak. This implies an autonomic control of the cardiovascular system with a preserved baroreflex function. Consequently, the cardiac output remains stable despite blood pressure decreases [26].

Our study was not concepted to compare the efficacy of IV versus PO nimodipine to prevent DCI or poor functional outcome because our patient population significantly differs in terms of disease severity and hospital complications reflecting our standard of care procedures in patients with SAH. There remains some controversy over the formulation in which nimodipine should be given. A recent meta-analysis showed that enteral and intravenous nimodipine may have a similar effectiveness in the prevention of poor outcome and DCI [5]. Some studies suggest more stable nimodipine concentrations with IV formulation [27] or a lower bioavailability of enteral nimodipine [28] which was not supported by others [29]. It is reasonable that continuous IV nimodipine application could smooth blood pressure control and avoid blood pressure fluctuations, which are known to be associated with DCI and poor functional outcome in patients with SAH [30, 31].

Some limitations to the study merit consideration. Firstly, we did not correct for other blood pressure lowering medications. However, as already mentioned it is unlikely that they were given concomitantly with nimodipine. Secondly, we did not consider CYP 3A4 inhibitors such as erythromycin, azole antimycotics, and valproic acid that can lead to increased plasma concentrations of nimodipine. Thirdly, we report two distinct groups of patients, which makes any comparisons impossible. Fourthly, we did not include patients with discontinuous blood pressure measurements to achieve a high resolution of blood pressure readings. This approach leads to a selection bias toward poor-grade patients and renders our results speculative for patients with good-grade SAH.

## Conclusions

Our study depicts time-locked blood pressure evolutions secondary to IV and PO nimodipine applications in patients with SAH. Our results show that although blood pressure drops were common, especially after IV nimodipine delivery in patients with poor-grade SAH, hypotensive episodes could have been prevented. Further randomized controlled studies are needed to test the effect of PO in comparison with IV nimodipine formulations on outcomes in patients with SAH aiming at avoiding blood pressure instability by integration of continuous blood pressure monitoring in the study protocol.

## Supplementary Information

Below is the link to the electronic supplementary material.

Supplementary file 1. Flow chart demonstrating patient selection (PDF 12 kb)

Supplementary file 2 (DOCX 49 kb)

Supplementary file 3. Median heart rate (HR) over time after oral nimodipine administration in the subgroups of intakes with (n=56/598, 9%) and without a significant >10% HR increase (TIF 1471 kb)

Supplementary file 4 (DOCX 14 kb)

Supplementary file 5 (DOCX 17 kb)
