# Supplementary figures and images for: Blood Pressure Changes in Association with Nimodipine Therapy in Patients with Spontaneous Subarachnoid Hemorrhage
Source: Neurocrit Care. 2023 Jun 12;39(1):104–15. doi: 10.1007/s12028-023-01760-y (PMC10499738; doi:10.1007/s12028-023-01760-y)

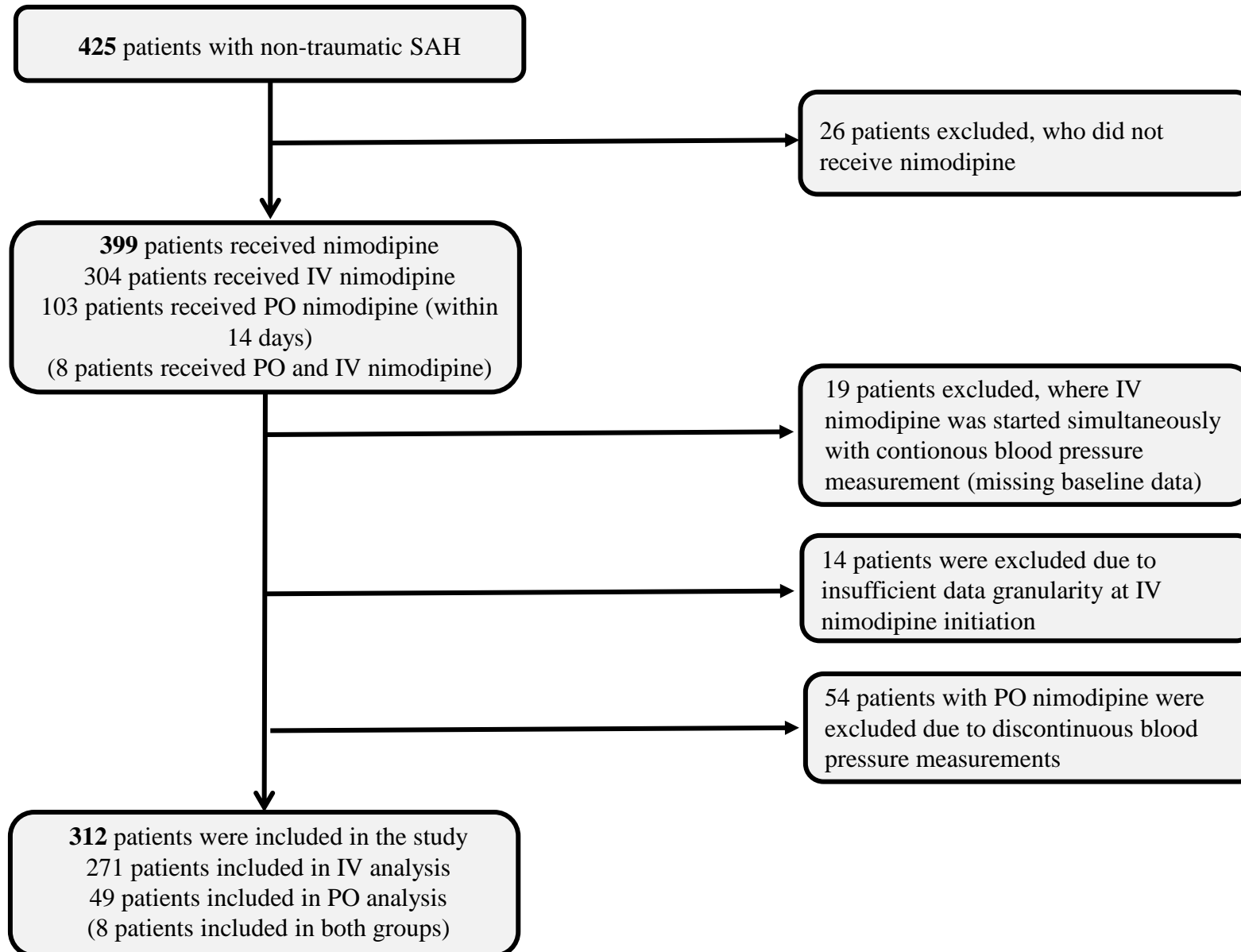

Supplement: Supplementary file 1 — Flow chart demonstrating patient selection (PDF 12 kb) [file 12028_2023_1760_MOESM1_ESM.pdf]
