# Supplementary material for: Blood Pressure Changes in Association with Nimodipine Therapy in Patients with Spontaneous Subarachnoid Hemorrhage
Source: Neurocrit Care. 2023 Jun 12;39(1):104–15. doi: 10.1007/s12028-023-01760-y (PMC10499738; doi:10.1007/s12028-023-01760-y)
Supplement: Supplementary file 2 — (DOCX 49 kb) [file 12028_2023_1760_MOESM2_ESM.docx]

**Supplemental Figure 2**


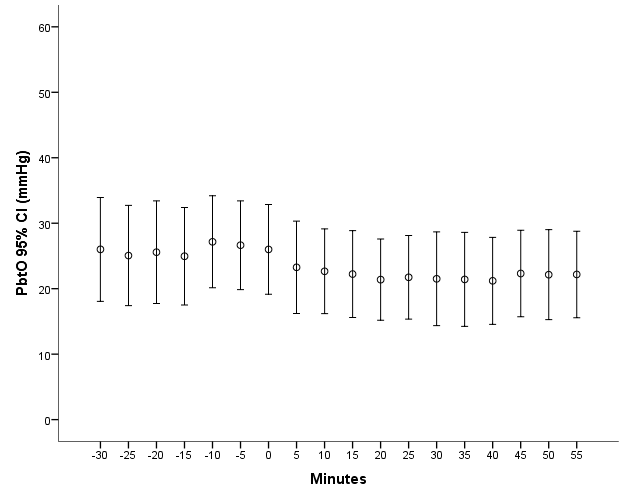


**B**

**A**


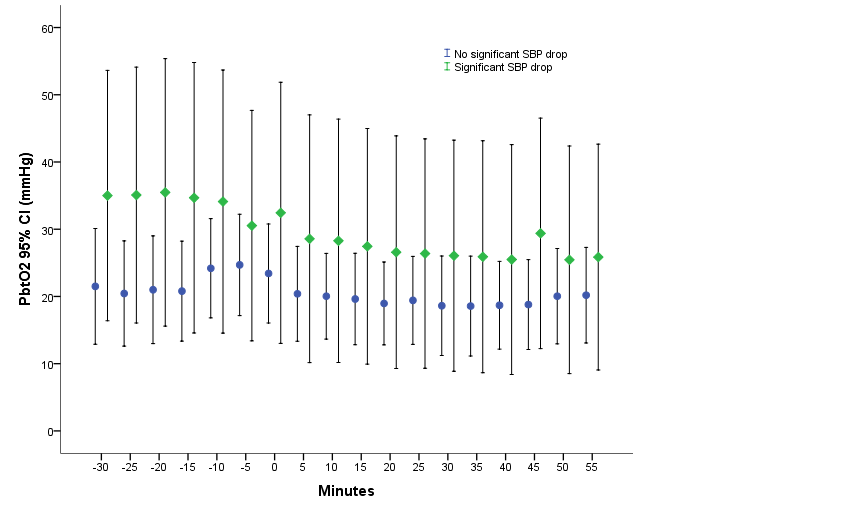


**Subanalysis of P_bt_O_2_ values before and after initiation of IV nimodipine in 22 patients.**

A) Overall, baseline (30 minutes before IV nimodipine start) P_bt_O_2_ was 25 ± 15 mmHg and decreased to 22 ± 14 mmHg (p=0.012; paired t-Test), indicating that mean P_bt_O_2_ levels were always above the critical threshold of 20 mmHg. B) When splitting patients based on a significant drop of SBP (n=7/22, 32%) vs. stable SBP levels after IV nimodipine initiation (n=15/22 68%), a significant P_bt_O_2_ drop was only found in the first group (baseline P_bt_O_2_: 32 ± 19 mmHg; mean P_bt_O_2_ within one hour after nimodipine start: 26 ± 19 mmHg, p=0.006). In comparison, patients with stable SBP levels after IV nimodipine initiation had stable P_bt_O_2_ levels (baseline P_bt_O_2_ 21 ± 13 mmHg; mean P_bt_O_2_ within one hour after nimodipine start: 19 ± 11 mmHg; p=0.218). On a statistical basis, baseline, and one-hour P_bt_O_2_ levels were similar across the two groups (p=0.162; p=0.407; resp.; Mann Whitney U Test).
