# Supplementary material for: Blood Pressure Changes in Association with Nimodipine Therapy in Patients with Spontaneous Subarachnoid Hemorrhage
Source: Neurocrit Care. 2023 Jun 12;39(1):104–15. doi: 10.1007/s12028-023-01760-y (PMC10499738; doi:10.1007/s12028-023-01760-y)
Supplement: Supplementary file 4 — (DOCX 14 kb) [file 12028_2023_1760_MOESM4_ESM.docx]

| Supplemental Table 1. Treatment intensification within one hour after IV or PO nimodipine application based on significant blood pressure drops. | | | | |
| --- | --- | --- | --- | --- |
| **IV group** | **All, n (%)**  **N=271** | **SBP drop >10% N=81** | **SBP stable N=190** | **P-Value*** |
| Group 1 (No treatment change or fluids only) | 125 (50) | 46 (57) | 89 (47) | 0.146 |
| Group 2 (Noradrenaline increase >10%** or start; with or without fluids) | 136 (50) | 35 (43) | 101 (53) |  |
| **PO group** | **All, n (%) N=601 intakes in 49 patients** | **SBP drop >10% N=53** | **SBP stable N=548** | **P-Value*** |
| Group 1 (No treatment change or fluids only) | 586 (97) | 51 (96) | 535 (98) | 0.634 |
| Group 2 (Noradrenaline increase >10%** or start; with or without fluids) | 15 (3) | 2 (4) | 13 (2.4) |  |
| Data are given in n (%).  * Differences across significant blood pressure drops vs none were calculated with the Fisher's exact test.  ** The noradrenaline dose one hour before to one hour after nimodipine application was compared. | | | | |
