# Supplementary material for: Blood Pressure Changes in Association with Nimodipine Therapy in Patients with Spontaneous Subarachnoid Hemorrhage
Source: Neurocrit Care. 2023 Jun 12;39(1):104–15. doi: 10.1007/s12028-023-01760-y (PMC10499738; doi:10.1007/s12028-023-01760-y)
Supplement: Supplementary file 5 — (DOCX 17 kb) [file 12028_2023_1760_MOESM5_ESM.docx]

| Supplemental Table 2. Univariate Analysis: Patient characteristics based on systolic blood pressure (SBP) drops after IV or PO nimodipine application. | | | |
| --- | --- | --- | --- |
| **IV group** | **SPB drop >10%**  **N=81** | **SBP stable**  **N=190** | **P-value*** |
| Age | 59 (53-70) | 58 (48-70) | 0.428 |
| Sex, female | 53 (65) | 128 (67) | 0.779 |
| Admission Hunt & Hess score | 3 (2-5) | 3 (2-5) | 0.078 |
| Modified Fisher Score on admission | 4 (2-4) | 4 (3-4) | 0.046 |
| Large-vessel vasospasm | 46 (57) | 102 (54) | 0.690 |
| Delayed cerebral ischemia | 19 (24) | 39 (21) | 0.629 |
| Poor 3-month functional outcome (modified Rankin Scale ≥3) | 36 (47) | 89 (51) | 0.586 |
| **PO group** | **SPB drop >10% (any per patient)**  **N=28 patients** | **SBP stable**  **N=21 patients** | **P-value*** |
| Age | 57 (49-67) | 51 (46-62) | 0.241 |
| Sex, female | 14 (50) | 10 (48) | 1.000 |
| Admission Hunt & Hess score | 1 (1-2) | 2 (1-2) | 0.580 |
| Modified Fisher Score on admission | 3 (1-3) | 2 (1-3) | 0.435 |
| Large-vessel vasospasm | 7 (25) | 5 (24) | 1.000 |
| Delayed cerebral ischemia | 1 (4) | 1 (5) | 1.000 |
| Poor 3-month functional outcome (modified Rankin Scale ≥3) | 2 (9) | 1 (5) | 1.000 |
| Data are given in n (%) or median (IQR).  * Differences across significant blood pressure drops vs none were calculated with the Fisher's exact test or Mann Whitney U Test. | | | |
